# Prevalence, patterns and predictors of paranormal beliefs in The Netherlands: a several-analysts approach

**DOI:** 10.1098/rsos.240049

**Published:** 2024-09-04

**Authors:** S. Hoogeveen, D. Borsboom, Š. Kucharský, M. Marsman, D. Molenaar, J. de Ron, N. Sekulovski, I. Visser, M. van Elk, E.-J. Wagenmakers

**Affiliations:** ^1^ Department of Methodology and Statistics, Utrecht University, Utrecht, The Netherlands; ^2^ Department of Psychology, University of Amsterdam, Amsterdam, The Netherlands; ^3^ Institute of Psychology, Leiden University, Leiden, The Netherlands

**Keywords:** paranormal beliefs, conspiracy beliefs, team science, many analysts

## Abstract

Paranormal beliefs encompass a wide variety of phenomena, including the existence of supernatural entities such as ghosts and witches, as well as extraordinary human abilities such as telepathy and clairvoyance. In the current study, we used a nationally representative sample ( N=2534 ) to investigate the presence and correlates of paranormal beliefs among the secular Dutch population. The results indicated that most single paranormal phenomena (e.g. belief in clairvoyance) are endorsed by 10–20% of Dutch respondents; however, 55.6% of respondents qualify as paranormal believers based on the preregistered criterion that they believe in at least one phenomenon with considerable certainty. In addition, we invited four analysis teams with different methodological expertise to assess the structure of paranormal beliefs using traditional factor analysis, network analysis, Bayesian network analysis and latent class analysis (LCA). The teams’ analyses indicated adequate fit of a four-factor structure reported in a 1985 study, but also emphasized different conclusions across techniques; network analyses showed evidence against strong connectedness within most clusters, and suggested a five-cluster structure. The application of various analytic techniques painted a nuanced picture of paranormal beliefs and believers in The Netherlands and suggests that despite increased secularization, subgroups of the general population still believe in paranormal phenomena.

## 1. Introduction

I assume that the reader is familiar with the idea of extra-sensory perception, and the meaning of the four items of it, viz. telepathy, clairvoyance, precognition and psychokinesis. These disturbing phenomena seem to deny all our usual scientific ideas. How we should like to discredit them! Unfortunately the statistical evidence, at least for telepathy, is overwhelming [1].

As highlighted in the epigraph, the founding father of computer science and artificial intelligence firmly believed in the reality of extra-sensory perception (ESP; [2]). In fact, the human ability for ESP constituted one of Alan Turing’s main arguments against the notion that machines can truly ‘think’. Turing’s conviction was strengthened, the story goes, by the card-guessing experiments of J. B. Rhine, which he regarded as providing ‘statistically overwhelming evidence’.^1^ Turing is not the sole famous scientist who was convinced of the existence of the paranormal. Sir Isaac Newton, the founding father of modern physics, was a devoted alchemist who experimented endlessly to discover the recipe for the mythical Philosopher’s Stone [4]; physicist Pierre Curie, husband of Nobel laureate Marie Curie, regularly attended séances of the (in)famous medium Eusapia Palladino [5]; and philosopher Arthur Schopenhauer argued that we cannot deny the evidence for predictive dreams and clairvoyance ‘now that countless testimonies, from the most credible circles, have confirmed such predictions of the future’ [6,7].

More recently, the continued popularity of gurus, shamans, faith healers and spiritual retreats (e.g. meditation or psychedelic retreats) reflects a continued and widespread interest in supernatural phenomena. This year several articles appeared in reputable newspapers about the hype surrounding ‘manifestation’: the ability to create your own reality by wanting, feeling and saying.^2^ According to a 2018 national survey among 1207 US respondents, approximately 75% of the American public believe in at least one of several paranormal phenomena such as psychic powers, haunted places, psychokinesis and alien visits ([8], for a review of previous surveys, see below).

First of all, it should be noted that the exact definition and demarcation of paranormal beliefs is far from unequivocal [9]. Paranormal beliefs are generally used as an umbrella term for a range of New Age beliefs, including belief in precognition, Psi, spiritualism, telepathy, psychokinesis, channelling, witchcraft and superstition. Some paranormal phenomena overlap with religious traditions, such as healing by the laying on of hands or reincarnation, while others are more related to everyday superstitions such as the alleged risk of walking under a ladder or the existence of lucky numbers. In the present paper, we adhere to the following working definition: paranormal beliefs are characterized by a violation of scientifically established natural laws of physics, biology and psychology [10], and are endorsed by people ‘who might normally be expected by their society to be capable of rational thought and reality testing’ [11, pp. 16–17]. In the narrow sense of the term, this definition excludes idiosyncratic false beliefs, including those attributed to mental disorders such as schizophrenia (i.e. delusions) because those are regarded as symptomatic of abnormal cognitive functioning [12]. Conspiracy beliefs and belief in pseudoscience such as homeopathy also fall outside of the current definition, as they do not violate natural laws in any way.^3^

Paranormal beliefs have been a topic of interest in the scientific literature. Specifically, many studies have investigated correlates of paranormal beliefs in the domain of cognitive functioning and personality characteristics. For instance, a wide variety of cognitive biases and reasoning errors have been associated with an increased tendency to believe in paranormal phenomena, ranging from agency detection biases [13–15], the illusion of control [11,16–18] and the self-attribution bias [19], to ontological confusions [10,20–23], illusory pattern perception [24–27] and jumping to conclusions [16,28,29]. Relatedly, a tendency to apply less stringent criteria for ‘evidence’ has been related to an increased likelihood to endorse paranormal beliefs; paranormal believers may be more likely to endorse many different statements, especially in the face of ambiguous information. In this regard, increased belief in paranormal phenomena has been correlated with susceptibility to suggestion [30], gullibility [31,32] and intuitive thinking as the overall dominant thinking style [22,33–41].

At the same time, relatively little is known about the prevalence of paranormal beliefs among the general public, as few studies with representative samples have been conducted. To the best of our knowledge, the few polls with representative samples mostly originate from the United States, a country with relatively high levels of religiosity and supernatural beliefs. For instance, in a 2005 survey, 37% of US respondents reported belief in the existence of cursed places (‘haunted houses’), 31% in telepathy and 20% in reincarnation [42].^4^ In 2018, these rates were even slightly higher; 58% of US respondents believed in places haunted by spirits, 26% in telekinesis (i.e. moving objects with mental force), 21% in the existence of Bigfoot and 17% in clairvoyance (i.e. predicting the future; [8]). A 2023 Ipsos survey showed similar rates; 39% of US respondents believed in ghosts, 42% in aliens who visited Earth as well as 34% in ESP and 22% in spells or witchcraft [43]. Overall, in both 2005 and 2018 about 75% of US respondents were classified as paranormal believers, based on the criterion that they endorsed at least one of the inquired paranormal phenomena [8,42].

Some further information is available about paranormal beliefs in the UK and Canada; according to a 2017 poll, 33% of the British believe in ghosts, ghouls, spirits or other types of paranormal activity, while 21% are unsure [44]. A Gallup poll from 2005 showed comparable levels of paranormal belief in the USA, Canada and the UK, with the most popular phenomenon in all three countries that of haunted houses. Additionally, Americans were somewhat more likely to believe in the existence of witches (21% versus 13% in Canada and the UK), whereas Britons were most convinced of the possibility to communicate with deceased (27% versus 21% in the USA and 24% in Canada; [45]).

How these rates compare to those in the non-English speaking world remains relatively unclear. A 1985 survey from the Netherlands—one of the world’s most secular countries—indicated more scepticism compared with current rates in The United States; about 40 years ago, 7% of the Dutch population believed in spirits and ghosts, 4% in contact with aliens, 15% in telekinesis, 11% in reincarnation (but also 32% in telepathy and 31% in clairvoyance, similar to or exceeding the US numbers (see table 6 in appendix A for the comparison of the 1985 and 2023 rates). It is unknown, however, how these rates have developed over the last 40 years. On the one hand, Dutch society has continued its path towards secularization, partly explained by an increasing focus on (scientific) education and higher social security [46]. This would suggest that the rate of supernatural beliefs, including paranormal beliefs, will have decreased since 1985. On the other hand, there are abundant media reports about the rise of a new form of spirituality, with elements of supernatural and conspiracy beliefs [47–50]. This modern movement has been referred to by scholars as conspirituality [51–54], in which belief in positive elements such as the power of love, connection and intuition go hand in hand with belief in negative external influences such as conspiracies about COVID vaccinations and underground paedophilia rings (e.g. Pizzagate). According to the reports by The Netherlands Institute for Social Research, the New Age movement indeed reached its peak of popularity in the 1980s [55,56]. In these reports, it is speculated, however, that later on, New Age beliefs were increasingly replaced by terms such as ‘new’, ‘holistic’ or ‘alternative’ spirituality, with a focus on intuition and individual feelings as the criterion for the truth. Although paranormal phenomena appear to have become more familiar among the general public throughout the last decades, it is unclear to what extent the Dutch actually believe in, let alone actively engaged with paranormal phenomena [55].

In the current study, we investigated the prevalence of paranormal beliefs among the Dutch population, using a representative sample of 2511 Dutch citizens. Respondents were presented with statements about 28 concrete paranormal phenomena, ranging from contact with aliens and spirits, to telepathy, reincarnation and lucky numbers. The study is in part a replication of the opinion survey entitled ‘secret forces’ conducted in 1985 by the Dutch Foundation for Statistics, complemented with items based on a recently developed paranormal belief scale [57]. The aims of the current paper are threefold: (i) we will outline the extent and pattern of paranormal belief in The Netherlands; (ii) we will evaluate predictors and correlates of paranormal beliefs based on the 1985 results and current literature, such as religious identity and conspiracy beliefs; and (iii) we will assess the replicability of the factor structure of paranormal beliefs reported in 1985. To achieve the last aim, we invited five independent teams of analysts with expertise in relevant methodology to provide their independent judgement. This so-called ‘several-analysts’ effort attempts to gauge the robustness of the conclusions across participating teams, thereby preventing the model myopia that accompanies the traditional publication model in which a single team conducts all of the analyses [58–60]. The present study is preregistered on the Open Science Framework (https://osf.io/jx758), where the questionnaire, the criteria used to identify ‘paranormal believers’ and ‘skeptics’, the planned analyses for the predictor evaluation, as well as the instructions to the analysis teams were defined before the start of data collection.

In addition to investigating the prevalence of paranormal beliefs in The Netherlands, we also sought to identify demographic predictors and general correlates of these beliefs. Based on the 1985 report and recent literature, we preregistered the following hypotheses regarding demographic predictors of paranormal beliefs:

1. Women report more paranormal beliefs than men [21,34,61].
2. People who identify as spiritual-but-not-religious (SbnR) report more paranormal beliefs than the other groups (spiritual-and-religious (S&R), religious-but-not-spiritual (RbnS ), not-religious-and-not-spiritual (nRnS); [10,62,63]).

In addition to these demographic characteristics, we also investigated two additional correlates of paranormal beliefs. First, research on religion has repeatedly shown that the strongest predictor of religiosity is exposure to credibility enhancing displays (CREDs): behavioural cues indicative of underlying beliefs, mainly from one’s parents and direct social network, such as engaging in prayer, attending religious services, wearing specific clothing, etc. [64–67]. In the current study, we adjusted existing CREDs items to capture parental acts related to belief in paranormal phenomena, such as attending paranormal fairs, engaging in paranormal activities (e.g. palm reading and seances) and speaking about paranormal encounters. This results in the third hypothesis:

3. Exposure to parental displays of belief in paranormal phenomena is positively associated with paranormal beliefs.

Additionally, both paranormal and conspiracy beliefs share the characteristic of being empirically unsubstantiated, yet concerning different domains (i.e. supernatural reality versus secret human activities). As illustrated by the phenomenon of conspirituality and supported by empirical research, paranormal beliefs are assumed to be positively related to conspiracy beliefs [68–71]. This leads to the fourth hypothesis:

4. Conspiracy beliefs are positively associated with paranormal beliefs.

In an exploratory analysis, we gauged the extent to which age, level of education and geographic location (large cities versus rest of the country) predict paranormal beliefs. Since the results of the 1985 Dutch survey contradict recent published findings in the literature, we did not preregister predictions for these variables.

### 1.1. Several-analysts approach

The final aim of this project was to investigate the (factor) structure of paranormal beliefs using different analytic techniques in a many-analysts fashion. A many-analysts approach involves different analysis teams that each attempt to address a particular research question using the same data but different statistical methods. This approach has been used to empirically assess analytic variability/robustness of research outcomes [60,72–81]. In the current project, we directly invited experts from research groups that have relevant knowledge concerning our statistical question and design (i.e. scale assessment). In particular, we recruited analysis teams specialized in latent variable modelling (i.e. confirmatory factor analysis), frequentist network analysis, Bayesian network analysis and latent class modelling. These techniques allow us to draw conclusions about the structure of paranormal beliefs, but also provide unique contributions and insights.

This more targeted form of a many-analysts approach we term a *several-analysts* approach (cf. [76]). Note that this approach differs from typical multi-analysts projects, since the explicit aim is to compare the outcomes from different methods, instead of quantifying variability naturally arising from executing different plausible analysis plans. In other words, rather than inviting as many analysts as possible and evaluating the differences in outcomes resulting from differences in analytic decisions made by the teams themselves, we intentionally invited teams with different methodological backgrounds and asked them to apply their specialty analysis to the data. The outcomes of our several-analysts approach should thus not be regarded as a natural consequence of different principled decisions. At the same time, the potential (lack of) variation in conclusions across analysis teams can be interpreted in terms of robustness; if all analytic techniques yield the same conclusions regarding the structure of paranormal beliefs, this increases our confidence about this structure, whereas vastly different conclusions will undercut this confidence.

The four participating teams were presented with two primary questions. First, *does the current dataset provide support for the four-factor structure identified in the 1985 study?* These factors were ‘extraordinary human abilities’, ‘supernatural reality’, ‘unearthly beings’ and ‘everyday superstition’, and the exact item loadings per factor are shown in table 1. Second, *what is the most important conclusion/finding based on these data and the application of the teams’ statistical technique?* The teams’ answers to these questions are presented in §3. A more extensive by-team analysis can be found in the electronic supplementary material (https://osf.io/645tz/).

**Table 1.** Factor analysis results regarding paranormal beliefs in The Netherlands measured in 1985 ( N=813 ).

		dimension			
item	description	I	II	III	IV
dimension I: extraordinary human abilities					
5	healing by laying on of hands	++			
8	life course description by possessions	++			
11	graphology (characterization by handwriting)	++			
13	dowsing (locating ground water using a rod)	++			
9	telepathy (mind reading)	++			
15	causing events by wanting (‘manifestation’)	++	+	+	
7	clairvoyance (seeing into the future)	++	+		
10	seeing events happening (while absent)	++	+		
dimension II: supernatural reality					
1	predictive dreams		++		
2	contact with deceased		++	+	
6	palmistry (palm reading)	+	++	+	
3	astrology	+	++	+	
17	reincarnation		++	+	
23	recounting from a previous life		++	+	
14	telekinesis (moving objects with mental force)^a^	?	?	?	?
dimension III: unearthly beings					
4	spirits and ghosts		++	++	
16	gnomes and elves		+	++	
12	contact extraterrestrial beings (aliens)			++	
24	devil possession			++	
18	sorcery, black magic	+		++	
19	haunted places		+	++	
dimension IV: everyday superstition					
22	misfortune from walking under a ladder			+	++
20	lucky numbers				++
21	unlucky numbers				++

*Note:*. ‘++’ Indicates factor loadings greater than 0.50 and ‘+’ indicates factor loadings between 0.40 and 0.49.

^a^ Item 14 on telekinesis was mentioned under dimension II but omitted from the factor loading table in the original document.

## 2. Methods

### 2.1. Participants

Data were collected on behalf of Stichting Skepsis (https://skepsis.nl/), a Dutch foundation dedicated to the promotion and practice of scientific scepticism, in collaboration with Kieskompas, an independent research agency specialized in polls for elections and scientific research.^5^ Participants were invited from the Kieskompas VIP panel, a stratified sample based on gender, age, level of education, migration background, Nielsen district^6^ and voting behaviour. Subsequently, the data were weighted based on the same characteristics so as to generalize the results to the Dutch adult population (analyses reported below are based on the weighted data). Data were collected between 23 April and 4 May 2023. In total, the sample included data from 2534 subjects (50.7% male, mean age = 49.91, s.d. = 18.06).

### 2.2. Sampling plan

We preregistered a sample size of around 2500, which allows for a 95% confidence interval with a less than 2% margin of error in the prevalence rates of paranormal belief.

### 2.3. Materials

The study used a cross-sectional design and included a variety of self-report measures. All measures were administered in Dutch.

#### 2.3.1. Paranormal beliefs

The main measure of interest concerns a paranormal beliefs scale. The questionnaire is primarily based on a 1985 survey conducted by the Dutch Foundation for Statistics (Nederlandse Stichting Statistiek; NSS). We considered this survey a suitable starting point because of (i) its suitability for the Dutch context, as the items were also developed for The Netherlands; (ii) the fact that the items are specific rather than general, which contributes to their validity by reducing context-sensitivity and reference group effects [82]; (iii) availability of the factor structure results that could be subjected to a ‘replication’, in addition to comparing the mere descriptive pattern; and (iv) existence of the 1985 belief rates, allowing for a (descriptive) historical comparison. Items 1–24 are taken directly from this survey. Items 25–27 are taken (in slightly adapted form) from the scale by Dean *et al*. [57] to complement the 1985 survey. Together, the final survey covers 11 of the 13 items from the recent and psychometrically validated scale by Dean *et al*.^7^ Item 28 was added based on the personal judgement and discussion; in particular, we added the item on the existence of benevolent spirits as counterparts to the existence of the evil spirits. All items were measured on a 5-point response scale ranging from ‘definitely not’ (1) to ‘definitely’ (5). The order in which the items were presented was randomized. A Bayesian reliability analysis using the Bayesrel package [83] indicated good internal consistency of the 28-item paranormal beliefs scale, McDonald ω = 0.964, 95% credible interval [0.962, 0.966].

#### 2.3.2. Conspiracy beliefs

The 5-item Conspiracy Mentality Questionnaire [84] was used to measure generic conspiracy beliefs. All items were measured on a 5-point response scale ranging from ‘definitely not’ (1) to ‘definitely’ (5). The presentation order of the items was randomized. A Bayesian reliability analysis indicated adequate internal consistency of the 5-item conspiracy beliefs scale, McDonald ω = 0.880, 95% CI [0.873, 0.888].

#### 2.3.3. Religious beliefs

Respondents indicated to what extent they identify with a religious community (0–100 scale); if identification was greater than 0, respondents additionally specified their religious group (Roman Catholic, Protestant, Muslim, Hindu, Jewish, Buddhist or other). Finally, respondents answered a self-categorization item classifying themselves as religious-and-spiritual (R&S), religious-but-not-spiritual (RbnS), spiritual-but-not-religious (SbnR) or not-religious-not-spiritual (nRnS).

#### 2.3.4. Parental paranormal affinity

Six items on parental affinity with the paranormal were included. Specifically, these items measured the extent to which one has observed parental behaviour related to belief in paranormal phenomena. This measure was based on the items used to assess credibility-enhancing displays of religion [64–66]. Here, we targeted parental acts related to paranormal beliefs, such as attending paranormal fairs, engaging in paranormal activities (palm reading and seances) and speaking about paranormal encounters. We created these items ourselves. Three items are measured on a 5-point frequency scale ranging from ‘never’ (1) to ‘often’ (5) and three items are measured on a 5-point degree scale ranging from ‘not at all’ (1) to ‘strongly’ (5). A Bayesian reliability analysis indicated adequate internal consistency of the 6-item paranormal CREDs scale, McDonald ω = 0.800, 95% CI [0.787, 0.812]. Dropping any of the items did not improve the estimated reliability.

#### 2.3.5. Additional paranormal items

For exploratory purposes, we additionally included: (i) a single item paranormal experiences to measure the extent to which participants had any personal paranormal experiences (5-point scale); (ii) single item paranormal beliefs to measure the extent of belief in paranormal phenomena (0–100 scale); (iii) single item parental paranormal beliefs to measure the extent of belief in paranormal phenomena by parents (0–100 scale); and (iv) single item paranormal beliefs prevalence estimation: estimate of percentage of the Dutch population that believes in paranormal phenomena.

#### 2.3.6. Quality check items

We included four items to identify mischievous survey respondents [85–88]. Three items use reports of low-incidence events as a flag for dishonest responding (i.e. implausible height, number of children and average hours of sleep) and one item directly asks whether participants engage in humorous or insincere responding (5-point). Participants were flagged as mischievous respondents if they reported implausible answers to at least two of the three low-incidence items or/and report to respond insincerely at least sometimes (i.e. a score of at least 3 on the 5-point Likert scale). Following Lopez & Hillygus [87], criteria for the low-incidence items were: (i) sleeping more than 11 h per day on average^8^; (ii) having more than seven children; and (iii) rare height (i.e. below the gender-specific 0.6th percentile or above the 99.4th percentile). Previous work used a criterion of the bottom or top 2% by gender [87]. We decided to be slightly more conservative and use the ±2.5 s.d. criterion (i.e. 0.6% and 99.4%). Based on the Dutch population, this means that for men the cut-off values were less than 166 and greater than 208 cm and for women less than 155 and greater than 187 cm [90]. As preregistered, we retained all observations for the primary analyses, but intended to investigate the effect of excluding cases flagged as mischievous respondents (but see below).

#### 2.3.7. Demographics

The following items were included: gender, age, level of education (eight levels) and perceived socioeconomic status (10 levels). Nielsen district was added based on the subjects’ province and municipality of residence. These last two pieces of data have been removed from the public dataset in order to safeguard anonymity.

### 2.4. Procedure

Respondents received a link to the Qualtrics survey and filled it out online. After giving informed consent, respondents provided demographics and completed the three low-incidence quality check items and the religiosity items. Then the 28 paranormal beliefs items were completed, followed by the conspiracy beliefs items, the parental affinity items, the additional single-items on paranormal beliefs and the final quality check item on humorous responding.

### 2.5. Data analysis

All results reported below (including figures and tables) are based on the weighted data unless indicated otherwise. The confirmatory analyses were conducted with a Bayesian linear regression model with gender (two levels), religious-spiritual identification (four levels), parental affinity score and conspiracy beliefs score as predictors and paranormal beliefs score (mean of 28 items) as the dependent variable. The exploratory predictors (i.e. age, level of education and location (large cities versus the rest)) were added to the same model. The BAS package [91] in R was used to conduct the linear regression. The Jeffreys–Zellner–Siow (JZS) prior was used for the coefficients and a beta-binomial (1,1) prior for the models. Order constraints were applied to test the predicted direction of the preregistered effects. Finally, to test the prediction that the single-item paranormal beliefs measure is strongly correlated with the overall scale ( r>0.7 ), we conducted a Bayesian Pearson correlation using the BayesFactor package [92]. Evidence is quantified by Bayes factors in the form of a ratio of the likelihood of the data under the hypothesis of interest versus the null hypothesis, indicated by subscripts. For instance, BF_+0_ is used in the case of a hypothesized positive effect for the reference group or a positive relation between variables; BF_10_ is used for an undirected effect (i.e. a two-sided hypothesis).

A short description of the methods used in the several-analysts component is included in the relevant section in §3. More details about the analytic techniques can be found in the electronic supplementary material (https://osf.io/645tz/).

## 3. Results

### 3.1. Descriptive results

**Figure 1.**
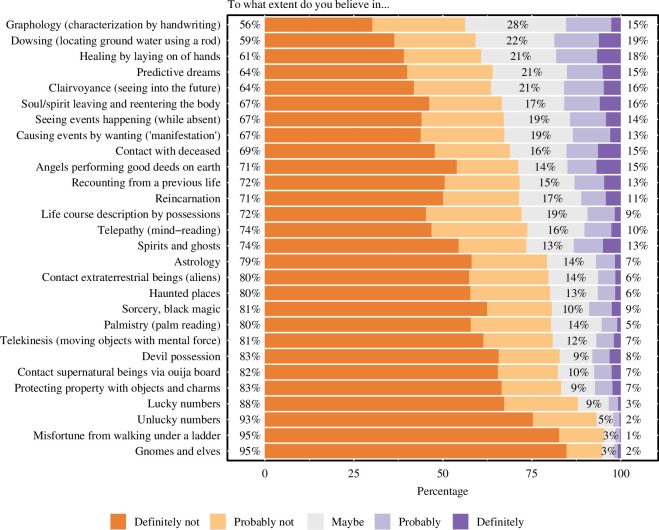
Endorsement of paranormal phenomena. Phenomena are ordered by average belief score (from high to low). The percentages listed reflect the proportion of disbelief, neutrality and belief for each phenomenon (from left to right).

The main dataset consisted of the 28 items of the paranormal beliefs scale. Figure 1 provides a visualization and summary of results per item of the paranormal beliefs scale (see also table 2 for the descriptives of the main measures in the study, and table 6 in appendix A for a comparison of rates of paranormal beliefs reported in 1985 and 2023). For the overall scale (i.e. average of the 28 items per subject), we find the following statistics: N= 2511, mean = 1.88 (s.d. = 0.77), median = 1.70 (mean absolute deviation (MAD) = 0.77), range: [1, 4.71].

**Table 2.** Descriptive statistics of the main measures in the study.

	mean	median	s.d.	min	max
paranormal beliefs	1.88	1.70	0.77	1.00	4.71
CREDs paranormality	1.25	1.00	0.44	1.00	4.50
conspiracy beliefs	2.89	2.80	1.02	1.00	5.00

*Note*. All items were measured on a 5-point Likert scale.

CREDs, credibility-enhancing displays.

In this sample, we identified 55.6% of paranormal believers according to the preregistered criterion that they believe in at least 1 of the 28 paranormal phenomenon with some certainty (i.e. a score of ‘4’ or ‘5’ on at least one item). In addition, we identified 18.6% sceptics according to the criterion that they indicated disbelief with some certainty on all 28 paranormal items (i.e. a score of ‘1’ or ‘2’ on all items). Interestingly, when using the continuous overall self-rated paranormal beliefs scale, 30.7% of respondents consider themselves sceptics, as they indicated 0 on the 0–100 scale. However, only 13.3% of respondents qualified as sceptics according to the criterion for the 28-item scale as well as the criterion for the continuous self-report item.

Finally, we asked respondents to estimate the percentage of the Dutch population endorsing paranormal phenomena. This was estimated, on average, at 35.7%—higher than the average self-reported belief on the 0–100 scale (i.e. 26.1) but lower than the 55.6% based on the criterion that one qualifies as a paranormal believer when one believes in at least one paranormal phenomenon with some certainty.

**Figure 2.**
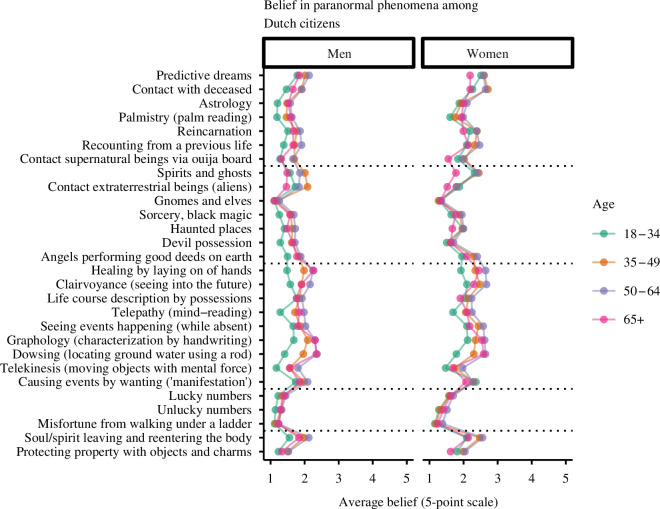
Average belief in paranormal phenomena by gender and age group. The items are grouped by the four categories identified in the 1985 survey. The bottom two items were added to the questionnaire and did not fit the existing categories. Belief was measured on a 5-point Likert scale ranging from definitely not to definitely.

Zooming in on the individual items highlights that the majority of the Dutch population is sceptical about all phenomena. At the same time, most paranormal phenomena were nevertheless considered ‘probably’ true or ‘definitely’ true by 10–20% of the people. As figure 1 shows, clairvoyance, healing by laying on of hands, the existence of angels and of spirits and ghosts are relatively popular paranormal beliefs. The traditional paranormal act of dowsing is endorsed by 1 in 5 respondents, slightly more than the spiritual trend of manifestation. Daily superstitions such as (un)lucky numbers and the danger of walking under a ladder were considered the least likely. Elves and gnomes induced even more scepticism and dangle at the bottom of the list—as they did back in 1985. A visualization of the average belief score per item split by age group and gender is given in figure 2.

#### 3.1.1. Conspiracy beliefs

**Figure 3.**
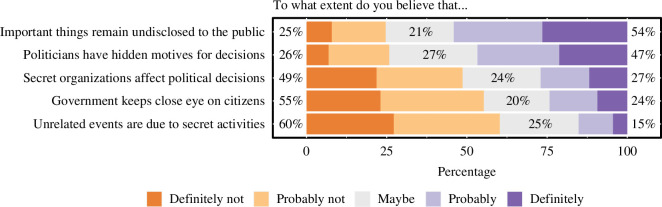
Endorsement of conspiracy beliefs. Statements are ordered by average belief score (from high to low). The percentages listed reflect the proportion of disbelief, neutrality and belief for each statement (from left to right).

Figure 3 shows respondents’ answers to the five conspiracy items. It appears that general conspiracy beliefs are more popular than paranormal phenomena.

#### 3.1.2. Mischievous responders

Our sample contained ‘only’ 2.50% of participants (i.e. 65 respondents) flagged as mischievous respondents, whereas previous work reported rates of 21% and 22% for conspiracy beliefs [87]. Moreover, the pattern of responses regarding the endorsement of paranormal beliefs was inconsistent; although for some items we found that participants flagged for mischievous responding were more likely to state that they ‘definitely’ believe in paranormal phenomena, for other items proportions were similar or even lower. This difference in rate and pattern of responses compared with previous work may partly be due to the topic (paranormal beliefs versus conspiracy beliefs), but we also believe our sample was of relatively high quality, as respondents were part of an existing opt-in panel, participated voluntarily and seemed to have taken the survey seriously. Since the rate of mischievous responders in the current study was low, we did not further analyse the data after removing the ‘trolls’ from the data but kept all observations as preregistered.

### 3.2. Confirmatory results

In line with the hypotheses we found that (see also table 3):

**Table 3.** Model-averaged posterior summary for regression coefficients of the paranormal beliefs data, using weighted regression.

coefficient	mean	s.d.	P (incl)	P (incl|D)	BF_incl_	lower	upper
intercept	1.884	0.012	1.000	1.000	1	1.862	1.907
woman	0.224	0.024	0.250	1.000	10^18^	0.177	0.270
R&S	−0.008	0.025	0.250	0.156	0.06	−0.092	0.001
RbnS	−0.535	0.038	0.250	1.000	10^40^	−0.609	−0.462
nRnS	−0.700	0.028	0.250	1.000	10^106^	−0.757	−0.646
CREDs	0.377	0.027	0.250	1.000	10^38^	0.322	0.431
conspiracy	0.212	0.012	0.250	1.000	10^60^	0.187	0.235
age (decades)	0.024	0.007	0.500	0.982	56.01	0.010	0.039
education	−0.002	0.006	0.500	0.204	0.26	−0.019	0.000
big cities	−0.003	0.015	0.500	0.128	0.15	−0.051	0.005

*Note*. The leftmost column denotes the predictor. The columns ‘mean’ and ‘s.d.’ represent the respective posterior mean and standard deviation of the parameter after model averaging. *P*(incl) denotes the prior inclusion probability and *P*(incl|D) denotes the posterior inclusion probability. The change from prior to posterior inclusion odds is given by the inclusion Bayes factor (BF_incl_). The last two columns represent a 95% central credible interval (CI) for the parameters. Order restrictions have been applied to the prior and posterior probabilities and Bayes factors where appropriate.

1. Women indeed reported more paranormal beliefs than men ( $M_{\text{women}}=$ 2.01, s.d._women_ = 0.83; $M_{\text{men}}=$ 1.67, s.d._men_ = 0.65; BF_+0_ = 10^18^).
2. People who self-identified as SbnR scored higher on paranormal beliefs ( $M_{\text{SbnR}}=$ 2.45, s.d._SbnR_ = 0.82) than those who self-identified as either RbnS ( $M_{\text{RbnS}}=$ 1.77, s.d._RbnS_ = 0.65), BF_+0_ = 10^40^ or as nRnS ( $M_{\text{nRnS}}=$ 1.48, s.d._nRns_ = 0.53), BF_+0_ = 10^106^. However, for the comparison between SbnR and R&S ( $M_{\text{R\textbackslash{}§amp;S}}=$ 2.25, s.d._R&S_ = 0.81), the Bayes factor indicated substantial evidence against the hypothesis: BF_+0_ = 0.064; BF_0+_ = 15.710.
3. Credibility-enhancing displays of paranormal beliefs observed from one’s parents were indeed positively associated with paranormal beliefs: BF_+0_ = $10^{38}$ . The model-averaged unstandardized regression coefficient of CREDs on paranormal beliefs is 0.377.
4. Conspiracy beliefs were also positively associated with paranormal beliefs: BF_+0_ = $10^{60}$ . The model-averaged unstandardized regression coefficient of conspiracy beliefs on paranormal beliefs is 0.212.

**Figure 4.**
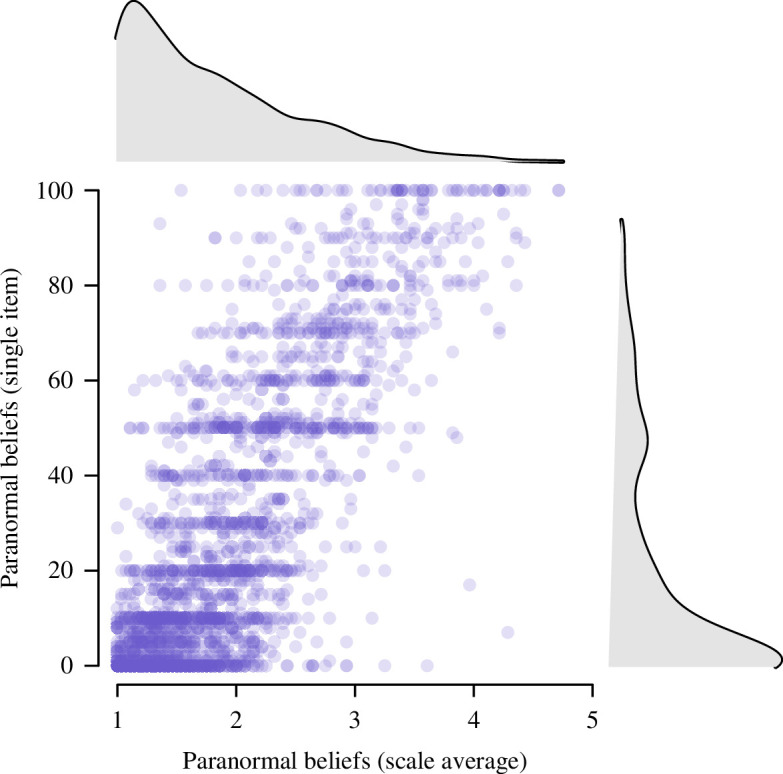
Correlation between the single-item self-reported paranormal beliefs (*y*-axis) and the average of the 27 paranormal belief items (*x*-axis), with the marginal density distributions on the sides.

Additionally, as preregistered, we tested whether the single-item paranormal beliefs measure was strongly correlated with the overall scale ( r > 0.7). We indeed found that the single-item paranormal beliefs measure (0–100 continuous scale) was strongly correlated with the overall paranormal beliefs score: ρ= 0.81, 95% CI [0.80, 0.83], ${\text{BF}}_{+0}=\infty$ .^9^ A scatterplot of the single-item paranormal beliefs measure and scale average is shown in figure 4.

### 3.3. Exploratory results

In an exploratory analysis, we examined the extent to which age, level of education and geographic location (large cities versus the rest) predict paranormal beliefs. Since the results of the 1985 survey contradict recent findings, we did not preregister a prediction for these variables and hence conducted undirected tests.

The data indicated that age was related to paranormal beliefs: BF_10_ = 56.015; older people tend to express more belief in paranormal phenomena (on average, paranormal beliefs increase with 0.02 on the 5-point scale with every 10 years). Level of education was not related to paranormal beliefs: BF_10_ = 0.256, nor was geographic location specified as living in the large cities versus the rest of the country: BF_10_ = 0.147.^10^

### 3.4. Several-analysts results

In the following, we provide the results and conclusions that the four different analysis teams provided regarding the (factor) structure of paranormal beliefs and the extent to which the factor structured reported in the 1985 survey could be replicated. In addition, the teams presented their most important insight based on their analysis.

These analyses are based on the first 24 items from the paranormal beliefs scale. Details on the methods and analyses can be found in the electronic supplementary material (https://osf.io/645tz/).

#### 3.4.1. Traditional factor analysis

##### Author: Dylan Molenaar

Two main models are considered: the first, referred to as ‘Theoretical Model’, is a four-dimensional factor model based on the theoretical structure underlying the 1985 analysis by the Dutch Foundation for Statistics. In the 1985 analysis, 16 cross-loadings were added to the theoretical model (see table 1), we refer to this model (including the cross-loadings) as the ‘Empirical Model’.

The Empirical Model and the Theoretical Model are fit to the 24 items of the paranormal beliefs scale using diagonally weighted least squares estimation in lavaan [94]. Model fit is judged by the root mean square error of approximation (RMSEA), the comparative fit index (CFI), and the Tucker–Lewis index (TLI) using the guidelines by Schermelleh-Engel *et al*. [95]. That is, for the RMSEA, values above 0.08 are taken as an indication of poor model fit, values between 0.05 and 0.08 are taken as an indication of acceptable model fit and values below 0.05 are taken as an indication of good model fit. For the CFI and TLI, values smaller than 0.95 will be taken as an indication of poor model fit, values between 0.95 and 0.97 are taken as an indication of acceptable model fit and values larger than 0.97 are taken as indication of good model fit.

The results are in table 4. As can be seen, according to all fit indices, the fit of the Empirical Model is good. As the Empirical Model has already been modified by adding the cross-loadings, and as the model fit is already good, the Empirical Model is not modified any further. The Theoretical Model fits acceptable according to the RMSEA and good according to the CFI and TLI. Modification indices indicated four residual covariances to be a source of misfit. These residual covariances are added to the Theoretical Model. As can be seen from table 4, the resulting modified Theoretical Model fits similarly well to the data as the Empirical Model, but with fewer parameters.

**Table 4.** Traditional confirmatory factor analysis: model fit results.

model	scaled $\chi^{2}$	d.f.	npar	RMSEA	CFI	TLI
empirical model	3006.529	230	142	0.047	0.997	0.996
theoretical model	4085.909	246	126	0.057	0.995	0.995
+free *r*17,23	3634.547	245	127	0.053	0.996	0.995
+free *r*18,24	3297.622	244	128	0.050	0.996	0.996
+free *r*1,7	3010.528	243	129	0.047	0.997	0.996
+free *r*3,6	2823.794	242	130	0.045	0.997	0.997

*Note*. ‘npar’: number of parameters. *rx,y* denotes the residual covariance between item *x* and item *y*.

Therefore, the conclusion is that the factor structure of the 1985 study by the Dutch Foundation for Statistics can be replicated, but that the model contains many unnecessary modifications. The model without modifications but with four additional residual covariances performs equally well to the model by the Dutch Foundation for Statistics, while the latter contains many more modifications.

### 3.4.2. Network analysis

#### Authors: Jill de Ron & Denny Borsboom

**Figure 5.**
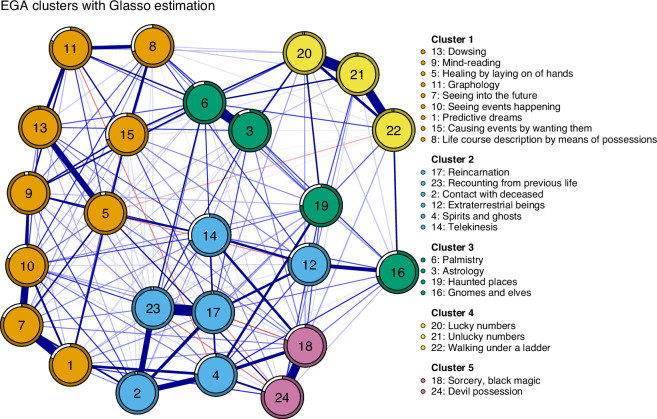
The network containing the 24 items of the paranormal beliefs scale. Blue edges represent positive partial correlations and red edges represent negative correlations; the greater saturation of the edge indicates a stronger partial correlation. The clusters were identified via a bootstrapped exploratory graph analysis with glasso estimation method. The pie chart around each node indicates the proportion that an item was placed in the same cluster out of 10 000 iterations of the bootEGA function. Most of the items had a high proportion (greater than 0.80) to be in the similar cluster across iterations.

Figure 5 shows the visualization of the unconstrained Gaussian Graphical Model containing the 24 items of the paranormal beliefs scale (estimated via the R package bootnet with Spearman’s correlations; [96]). As is typical for psychological networks, we found a dense network in which all nodes are connected to at least one other node, and with mostly positive edge weights. We used bootstrapped techniques from the bootnet package to assess the stability of the edge weights, which seem relatively stable (see electronic supplementary material; [97]).

To gain insight into the cluster structure of the network, we performed a non-parametric bootstrapped exploratory graph analysis with glasso estimation (bootEGA with 10 000 iterations; [96]). The bootEGA findings do not support the four-factor structure identified in the 1985 study. Instead, the most frequent number of clusters was five, which was observed in 5969 out of the 10 000 iterations. As the variables in the dataset are highly skewed, we also performed bootEGA with the TMFG estimation procedure, which resulted in three clusters (see electronic supplementary material, table S2). That different estimation procedures lead to different results indicates that the number of clusters is unclear.

The items featured consistent assignment to clusters, with a high proportion (greater than 0.80) being assigned to the same cluster across iterations (see electronic supplementary material, table S1). Both cluster 1 (‘extraordinary human abilities’) and cluster 4 (‘everyday superstition’) are similar to the factor structure found in the original 1985 study. However, the current analysis suggests that ‘predictive dreams’ should be assigned to cluster 1, which seems sensible as it represents an extraordinary human ability.

Within some of these clusters, there are clear evidential relations between the beliefs, with one being a necessary or sufficient condition for the other. One example is the strong partial correlation ( r=0.23 ) between the belief in ‘spirits and ghosts’ (item 4) and the belief in ‘contact with the deceased’ (item 2)—believing in the possibility of engaging in communication with the deceased necessitates the belief in ghosts and spirits, while the belief that one can communicate with the deceased is a sufficient condition for believing in ghosts and spirits. Another example is the strong connection between the three items ‘seeing into the future’ (item 7), ‘predictive dreams’ (item 1) and ‘seeing events happening’ (item 10), as believing in predictive dreams or direct perception of future events necessitates a belief in the ability to see into the future, while the latter is sufficient for the former. To the extent that the relevant associations are due to direct evidential relations rather than to the common influence of a latent variable, this suggests that a network representation may be preferable to a latent variable model for the relevant items.

### 3.4.3. Bayesian network analysis

#### Authors: Nikola Sekulovski & Maarten Marsman

**Figure 6.**
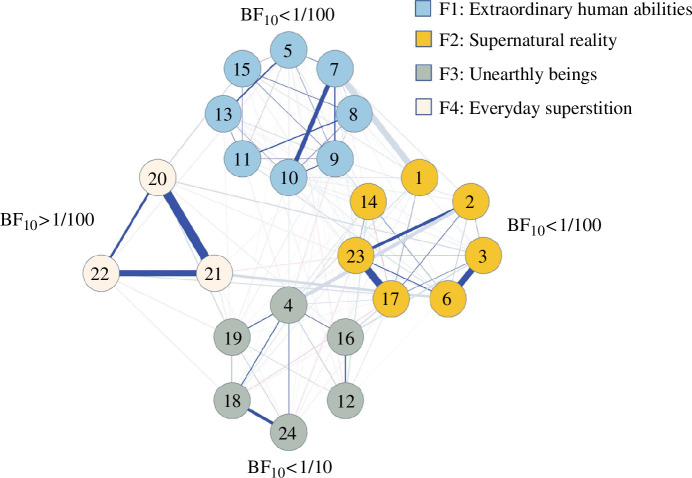
The estimated network structure represents the median probability model, which includes all edges with at least 0.5 probability of inclusion. The nodes are grouped based on the 1985 factor structure. Positive relations between items are depicted by blue edges, while negative relations are represented by red edges. Thicker edges indicate stronger relations. It is important to note that edges between clusters (i.e. factors) are intentionally less pronounced to place a stronger emphasis on the factor structure. Each cluster is accompanied by the BF_10_, which indicates the level of support for $H_{1}$ against $H_{0}$ .

The Bayesian analysis of the network structure [98,99], taking into account the factor structure obtained in the 1985 study, reveals insufficient evidence to support the original four-factor structure solution. We compare the cluster-level Bayes factors (BF_10_) for the hypothesis $H_{1}$ , which states that there is a direct connection between all pairs of items within a cluster, against a null hypothesis $H_{0}$ , which states that there is not a direct connection between all pairs of items within a cluster. Based on the results, only the BF_10_ for the fourth factor, ‘Everyday superstition’, which includes items 20, 21 and 22, shows support for $H_{1}$ . Conversely, the BF_10_ for the remaining three factors provide strong evidence against $H_{1}$ . It is worth noting that we used a cut-off of BF_10_ > 10 (or < 1/10; [98]); nevertheless, all four Bayes factors exceeded this threshold by a considerable margin. Our results are shown visually in figure 6. For the details of this analysis, we refer the reader to the electronic supplementary material.

### 3.4.4. Latent class analysis

#### Authors: Šimon Kucharský & Ingmar Visser

In principle, latent class analysis (LCA) cannot directly address the factor structure of the items; LCA searches for clusters of respondents that have something in common, whereas factors are clusters of items that have something in common. As such, LCA is not suitable to provide evidence for a factor structure of a questionnaire, as it answers a completely orthogonal question to that (cf. [100]). That said, we found some evidence that informing the LCA model (custom-coded in the probabilistic programming language Stan; [101]) with constraints that allow grouping the items into clusters based on the original confirmatory factor analysis (CFA) factors improves the fit of the model and finds additional groups of participants in the data; most prominently, a group of participants that are relatively sceptical towards everyday superstitions but less sceptical towards other types of paranormal phenomena. As such it is plausible that a four-factor model would fit the data, even though it is likely that the factors would be highly correlated, raising the question of whether it is practical to distinguish between the four factors in the first place. In fact, the interest in these data may lie more in the possibility to distinguish groups of people with various levels of scepticism, than in determining the commonalities between items.

The best-fitting model is informed by the four-factor structure found in 1985 and allows the agreement with each item to vary across items. This suggests the following:

– There is a substantial variability in agreement between items which is not captured properly if we constrain η across items.
– Allowing the model to classify participants into 16 groups (all combinations of the four factors) instead of two, while only allowing ‘low’/‘high’ responses on each item substantially improves fit to the data; this suggests that there are subgroups that score high on items associated with one factor but low on items associated with other factor(s).

In particular, the LCA indicates that about half (47%, 95% CI [45, 49]) of the population are extremely sceptical towards any kind of paranormal phenomena, uniformly across all item types. About a quarter of the population (25%, 95% CI [23, 28]) are less sceptical towards any and all paranormal phenomena. About a tenth of the population (10%, 95% CI [8, 11]) are extremely sceptical towards everyday superstitions, but less sceptical towards other types of paranormal phenomena. The proportions for each of the 16 estimated classes are shown in table 5. Note that in the table the second class is called ‘believers’ although the average score in this group was still below the midpoint of the scale, between ‘probably not’ and ‘maybe’—the term ‘open-minded-skeptics’ may be more appropriate than ‘believers’.

**Table 5.** Latent class analysis: proportions of the 16 classes estimated by the model.

		95% CI		
class	proportion	lower	upper	count
‘skeptics’	0.47	0.45	0.49	1302
‘believers’	0.26	0.23	0.28	668
EHA + SR + UB	0.10	0.08	0.11	212
EHA + SR	0.04	0.03	0.05	145
UB	0.02	0.02	0.03	41
EHA	0.02	0.01	0.03	66
ES	0.02	0.01	0.03	54
EHA + ES	0.01	0.01	0.02	27
EHA + UB	0.01	0.01	0.02	25
SR + UB + ES	0.01	0.00	0.02	5
EHA + SR + ES	0.01	0.00	0.02	21
SR + UB	0.01	0.00	0.01	11
SR	0.01	0.00	0.01	15
SR + ES	0.00	0.00	0.01	9
ES + UB	0.00	0.00	0.01	7
EHA + UB + ES	0.00	0.00	0.03	0

*Note:* EHA, belief in extraordinary human abilities; SR, belief in supernatural reality; UB, belief in unearthly beings; ES, belief in everyday superstition.

## 4. Discussion

The finding of 38 years ago that 40% of the Dutch population believe in dowsing and healing by laying on of hands was met with some surprise at the time. In our current replication of the survey, we found that most percentages have dropped considerably, in some cases by half or more: where in 1985 40% of respondents indicated that they believed in graphology, dowsing and healing by laying on of hands, this is now less than 20%. At the same time, using the criterion that one qualifies as a paranormal believer if one endorses at least one phenomenon with some certainty, more than half of the Dutch population (55.6%) could be considered a paranormal believer in 2023. We note that for two items, one could argue that they can be interpreted in a non-paranormal manner: (i) graphology—someone’s handwriting might give a clue about personality characteristics such as conscientiousness; and (ii) life course description by means of possessions—someone who owns a lot of soccer-related items is probably a soccer fan, and someone who owns a lot of toys is probably a child or is raising one. Nevertheless, after removing these two ambiguous items from the survey, the rate of believers remains relatively high at 52.5%.

In comparison with the 1985 data, it is notable that belief in phenomena surrounding the survival of the soul after death has remained stable: contact with the deceased, reincarnation and knowledge of a past life remains probable for 12–15% of Dutch citizens. In general, we see that belief in ‘positive supernatural forces’, such as prophetic dreams, is stronger than in negative forces, such as the existence of cursed places. Remarkably, belief in unearthly beings seems to have increased the most over the last 40 years: belief in contact with aliens, ghosts and spirits, and gnomes and elves has roughly doubled from 1985.^11^ Yet again, these rates are still considerably lower than those reported in the United States; both in terms of the endorsement rate for specific paranormal phenomena and the overall rate of paranormal beliefs based on believing in at least one phenomenon the Dutch are considerably more sceptical than the Americans, as well as the British and the Canadians [8,42–45]. For example, the existence of ghosts is believed by 39% of Americans versus only 13% of the Dutch. Based on the endorsement of 10 items, 75% of Americans qualify as paranormal believers, whereas this was 56% of the Dutch based on almost three times as many items.

Media reports on the rising popularity of paranormal and spiritual beliefs notwithstanding, our results are not particularly surprising in the context of the general perception of the Dutch as down-to-earth, sceptical people. Moreover, several recent studies investigating the prevalence of supernatural beliefs in The Netherlands paint a similar picture [56,102]. For instance, in a recent large-scale cross-cultural study, we found that The Netherlands was among the countries with the lowest reported levels of religiosity and that the Dutch were the most sceptical about the possibility of mental and physical states continuing after death (i.e. in the afterlife; [103,104]). Another study using a representative sample indicated that of those not believing in God, the majority of the Dutch were classified as analytic atheists, rejecting any type of supernatural reality or entities [105]. Finally, a recent study highlighted that in many (secular) countries, including The Netherlands, science constitutes the most important world-view among non-believers, suggesting a role for (scientific) education in the process of secularization [106].

In terms of the predictors of paranormal beliefs, we obtained strong evidence that gender, spiritual and religious identification, exposure to parental displays of paranormal affinity and conspiracy beliefs are related to paranormal beliefs; paranormal believers are more likely to be women, to consider themselves spiritual but not religious (or religious and spiritual), to have observed their parents engage in paranormal activities and to hold general conspiracy beliefs. Contrary to 1985, however, we found no evidence that young people are more drawn to the paranormal—on the contrary, age was positively, though weakly, related to paranormal beliefs. Notably, the 1985 youth group—today’s 56–62 year olds—is still the group with the strongest paranormal belief. This might suggest a cohort effect in 1985—as suggested in the 1985 report by the Dutch Foundation for Statistics and elsewhere [55]. Our data do not speak to what is driving this relatively high rate of paranormal belief among Generation X, but socio-historical and economic factors including secularization and economic uncertainty might have played a role. In general, the adaptive value of paranormal beliefs on a personal level may include a mix of social, motivational and epistemic aspects, contribute to a positive self-image, coping and provide a sense of control and meaningfulness [107–110], yet our data do not allow for elucidating such functional roles.

Based on the current findings and media reports, we could speculate that the tendency to appeal to a ‘different’ reality still remains prevalent, yet its main shape has changed. Instead of relying on the extraordinary abilities of some paranormally gifted individuals (e.g. palm readers, clairvoyants and mental healers), the modern focus seems to be on everyone’s own individual mental powers, such as manifesting one’s dreams and wellness-related pseudo-scientific claims to boost one’s health, but also conspiracy beliefs about secret organizations and pharmaceutical cover-ups. The current data also demonstrated that conspiracy beliefs are far more prevalent than paranormal beliefs (i.e. a mean score of 2.72 versus 1.83 on the 5-point Likert scale), with 54% of the Dutch believing that ‘Important things remain undisclosed to the public’. Although historical comparison data are lacking, it seems plausible that in contrast to paranormal beliefs, (general) conspiracy beliefs have in fact increased over the last decades, especially against the backdrop of the COVID-19 pandemic, which marked a rise in misinformation and conspiracism ([111,112], but see also [113,114]). We note, however, that the paranormal beliefs and conspiracy beliefs scales used in the present study are not comparable in nature, as the items on paranormal beliefs ask about specific, concrete phenomena (e.g. reincarnation and telepathy), whereas the conspiracy items target more general openness to the possibility of conspiracies (i.e. some politicians may have hidden motives).

This difference between scales also relates to an important limitation of the current study: our paranormal beliefs scale included very specific phenomena. While this set-up has the benefit of being concrete and straightforward to interpret for respondents, it also risks the possibility of missing relevant phenomena and being too culture- and time-specific. For instance, the act of dowsing and earth radiation to locate hidden water sources was relatively popular in 1985, but it was probably less familiar to the younger generations in our sample (see figure 2). In addition, the current findings are not directly generalizable beyond the Dutch population; both the included phenomena and measured belief rates are specific to the Dutch context. This limitation on generalizability mainly affects the prevalence rates and potentially the findings regarding the structure of paranormal beliefs and the identification of different groups of believers/sceptics. We believe the findings regarding the predictors and correlates of paranormal beliefs are more universal and have also been found in different countries [10,21,61,63,68–70].

At the same time, caution is warranted in interpreting demographic differences in paranormal beliefs, as previous work has demonstrated that observed differences could be inflated by differential item functioning (DIF; [115–117]). That is, gender differences, for instance, may be partly driven by differences in the perceived meaning of the items by men and women, rather than genuine differences in belief. While 11 of the 28 items included in the present paranormal beliefs scale largely overlap with the validated scale by Dean *et al*. [57], which did not show DIF, we cannot rule out that differences in wording may have induced DIF in the current items.

In addition to assessing the prevalence and correlates of paranormal beliefs, we also investigated the (factor) structure of paranormal beliefs. We invited four teams with different methodological expertise to examine to what extent the factor analytic results reported in a survey from 1985 using the same items could be replicated in the current data. The teams applied traditional confirmatory factor analysis, network analysis, Bayesian network analysis and LCA to address this question. The results revealed some support for the 1985 finding of four underlying factors of paranormal belief: extraordinary human abilities, supernatural reality, unearthly beings and everyday superstition. In particular, the model based on the 1985 report showed adequate fit according to the traditional confirmatory factor analysis, and informing the LCA by the 1985 factor structure did improve the model fit. At the same time, the four analytic techniques also indicated notable differences in the conclusions they come to based on the same underlying data. For instance, the frequentist network analyses suggested a preference for a five-cluster structure over the original four-cluster structure. Moreover, for three of the four original clusters, the assumption of all items within a cluster being strongly connected did not hold according to the Bayesian network analysis. Finally, the LCA identified different groups of people regarding their paranormal affinity, with almost half of the respondents qualified as general sceptics, followed by overall believers (though not necessarily strong believers, but rather open-minded sceptics) and those that reject superstition but are somewhat open to other paranormal beliefs.

These results paint a nuanced picture of paranormal beliefs among the Dutch population. Overall, the estimated network based on the frequentist analysis was quite dense, with almost exclusively positive relations between items, suggesting that the phenomena indeed share some general features. Yet the Bayesian network analysis revealed strong evidence against a direct connection between items within the clusters ‘extraordinary human abilities’, ‘supernatural reality’ and ‘unearthly beings’. That is, while some clusters of items could be identified and improved the model fit, the four-factor solution found in 1985 did not seem robust across all four analytic traditions. This interconnectedness of all items without clear sub-clusters also fits the descriptive pattern as well as the theoretical assumption of paranormal beliefs as an idiosyncratic, polymorphic system of supernatural beliefs; instead of prescribed religious doctrines, one can adhere to an individual, customized package of supernatural phenomena, with elements ranging from clairvoyance to witchcraft and contact with aliens to reincarnation. The descriptive pattern of the data similarly fits these ideas as we found belief rates of 10–20% for most items, with different people endorsing a selection of different items, instead of 10–20% of people endorsing the majority of items.

At the same time, some items display clear evidential relations, such as belief in spirits and ghosts being a necessary condition for believing in contact with the deceased, and the possibility of reincarnation being a necessary condition for believing in the ability to recount from a previous life. The presence of these direct evidential relations might render a network representation of paranormal beliefs more suitable than a latent variable model in terms of understanding the structure of paranormal beliefs. The main exception to the generality of the paranormal phenomena network may be the items on superstition (lucky/unlucky numbers and misfortune from walking under a ladder); these three items were consistently identified as a separate cluster, and also constituted the demarcation of two groups of people in the LCA: those who are slightly open to all paranormal phenomena (26%) and those who are open to most phenomena but highly sceptical towards everyday superstition (10%).

In general, we believe this study highlighted the value of the several-analysts approach in which specific methodological experts are invited to answer a given question; this set-up allows for unique insights that would not have been captured when consulting only one team with a specific background. At the same time, by asking a limited number of specific experts, the logistics and organization of the project remain manageable (for comparison: see [118], in which we discuss some challenges of a full multi-analyst project). Approaching a given research question with different methodologies provides a concrete demonstration of the robustness, nuance and unique angles of the results at hand. While this approach introduces more uncertainty in the outcomes and conclusions, it also generates a more complete and truthful picture of the data and by extension of reality [59,60,119,120].

A practical limitation of the several-analysts approach is that it requires the lead team to be aware of suitable methodological approaches and the associated experts; instead of letting the results show the variety of analytic approaches, one should estimate which approaches are relevant in advance. Moreover, some research questions might be more appropriate than others for inviting different methodological experts; a research question and design that clearly dictates a t -test might leave little room for different analytic strategies. At the same time, our experience with a previous multi-analyst project emphasized that the employment of different methodological or statistical approaches should not be understated; we expected mostly multi-level regression models in the Many-Analysts Religion Projects, yet were surprised to witness the variety of analyses, including machine learning, structural equation modelling, t -test, multiverse analysis and network analysis [81,118]. Furthermore, we believe the several-analysts design could also be extended to involve different *theoretical* experts, instead of solely methodological specialists, somewhat similar to an adversarial collaboration.

## 5. Conclusion

In conclusion, the overall level of paranormal beliefs in a representative sample in The Netherlands appears low; none of the inquired phenomena received support from more than one in five Dutch people. At the same time, more than half of the population endorses at least one paranormal phenomenon with some certainty. In addition, our several-analysts results indicated that a previously identified four-cluster structure of paranormal beliefs with ‘extraordinary human abilities’, ‘supernatural reality’, ‘unearthly beings’ and ‘everyday superstition’ may provide an adequate fit for the current data, yet may not unequivocally reflect the optimal structure to classify paranormal beliefs and believers. In general, we would advocate for this several-analysts approach as a practical alternative to full-blown multi-analyst projects that still enable one to empirically capture analytic robustness and uncertainty. By directly inviting a variety of experts, the approach strikes a balance between informativeness and effort, and may thus be realistically applicable across many empirical studies. As our demonstration emphasizes the several-analysts approach can offer different conclusions and take-home messages based on the same data, exposing contradictions between techniques, but also highlighting complementary insights.

## Figures and Tables

**Figure 7.**
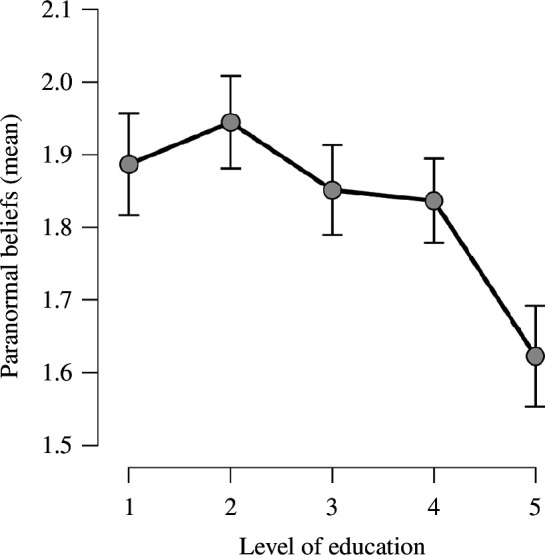
Descriptive plot of the relation between level of education and paranormal beliefs. Analysis and figure created in JASP using the BAIN module [129,130].

## Data Availability

The current study was preregistered on the Open Science Framework; readers can access the preregistration, as well as all materials for the study, the anonymized raw and processed data (including relevant documentation) and the R code to conduct all analyses (including all figures), on the OSF at [121]. Any deviations from the preregistration are highlighted in this manuscript. In particular, we preregistered inviting five teams of analysts. However, the expert on Bayesian factor analysis was unable to participate in the end. We therefore report the results of the four remaining analytic techniques.

## Footnotes

^1^
For a discussion on psychic experiments from the perspective of statistical evidence, see Wagenmakers *et al*. [3].

^2^
Or in the case of well-known YouTube gurus, Moneyfestation (tagline: *never want to worry about money again*? *Then start moneyfesting today*! Course fee: €199).

^3^
Irrespective of the evidence, homeopathy assumes that some physical substance can have a physical effect, which is in line with natural laws. Mental healing practices instead assume that the mental power of one individual can have a physical effect on another individual, which does conflict with natural laws as we currently understand them.

^4^
Only ‘channeling’—allowing a ‘spiritual being’ to temporarily take control of the body—was endorsed by ‘only’ 9%.

^5^
Both Skepsis and Kieskompas were not directly involved in this research or its outcomes.

^6^
Nielsen districts are regional classifications for the purpose of market research, dividing The Netherlands into five regions.

^7^
One additional item from Dean *et al*. [57] was included in the preregistration. This item on whether ‘mentions of a sixth sense are based on fantasy’ was excluded upon discussions with the Kieskompas survey experts, as it did not fit the answer options nor the overall question (‘To what extent do you believe in …’). The final item from Dean *et al*. on the soul surviving physical death was excluded as we considered it too prevalent in mainstream religious traditions to be included as a paranormal phenomenon.

^8^
We note that some research has shown that about 14% of the US population reports 9 or more hours of sleep, with up to 17.5% among those diagnosed with depression [89]. The prevalence of 11 h or more of sleep is not specified, however.

^9^
Given the positive skew in both the scale average and the single-item continuous rating, we also conducted a rank-based Spearman correlation test [93]. Again, the data indicated strong evidence for a strong correlation: 0.80, 95% CI [0.79, 0.81], BF_+0_ = 10^153^.

^10^
See appendix B for a further exploration of the relationship between education and paranormal beliefs.

^11^
Although we note that a change from 1% to 1.8% for belief in gnomes and elves should not receive too much weight.

## Appendix A: Paranormal beliefs in 1985 and 2023

Table 6 provides the rates of paranormal beliefs in The Netherlands reported in 1985 and 2023. Note that percentages were rounded to integers in 1985 whereas we included one decimal in the 2023 data. Four items were added in the current survey and hence do not have rates in 1985.

**Table 6.** Belief in paranormal phenomena in 1985 and 2023.

	year	
item	1985	2023
graphology (characterization by handwriting)	42%	14.8%
predictive dreams	22%	16.3%
clairvoyance (seeing into the future)	31%	17.3%
healing by laying on of hands	41%	17.5%
dowsing (locating ground water using a rod)	41%	15.9%
causing events by wanting (manifestation)	23%	15.6%
soul/spirit leaving and reentering the body	—	17.0%
seeing events happening (while absent)	34%	16.4%
contact with deceased	15%	15.9%
angels performing good deeds on Earth	−	16.6%
spirits and ghosts	7%	16.3%
reincarnation	11%	12.1%
recounting from a previous life	10%	13.3%
life course description by possessions	20%	9.5%
telepathy (mind reading)	32%	9.8%
contact extraterrestrial beings (aliens)	4%	8.5%
haunted places	8%	6.7%
sorcery, black magic	12%	11.2%
astrology	13%	7.0%
contact supernatural beings via ouija board	−	9.9%
palmistry (palm reading)	11%	5.7%
protecting property with objects and charms	−	9.6%
telekinesis (moving objects with mental force)	15%	6.7%
devil possession	10%	8.1%
lucky numbers	9%	4.4%
unlucky numbers	6%	2.6%
misfortune from walking under a ladder	4%	1.8%
gnomes and elves	1%	1.7%

*Note*. Percentages reflect the percentage of people answering ‘definitely’ (5) or ‘probably’ (4) on each item. Items are ordered according to the average belief score in 2023. Percentages are based on representative samples of *N* = 2610 respondents in 2023 and 813 respondents in 1985.

## Appendix B: Level of education and paranormal beliefs

The exploratory analysis on the predictors of paranormal beliefs as reported in the main text, indicated the absence of evidence for—or weak evidence against—a linear relation between level of education (eight levels) and paranormal beliefs score (BF_10_ = 0.256; BF_01_ = 3.90). We had not specified a directed hypothesis on the role of education, as the 1985 report found that especially the highly educated endorsed paranormal phenomena. Recent literature, on the contrary, typically reports an inverse relation between educational attainment and paranormal beliefs [61,122,123], although the relation tends to be weak and/or inconsistent [34,124]. As found for religiosity and pseudoscience [38,102,125–128], paranormal beliefs may be more strongly predicted by thinking style (i.e. preference) rather than thinking ability.

 In order to further explore the relation between education and paranormal beliefs in the current study, we delved deeper into the data. First, we realized that the lowest two levels were hardly present in the data, which is not surprising as very few people in The Netherlands do not complete some form of secondary education. We therefore combined the lowest three levels into one level, resulting in five groups of more similar size. The descriptive results as shown in figure 7 suggest that only the highest level of education (i.e. postdoctoral education) is associated with substantially lower paranormal belief, or perhaps a negative trend from levels 1 and 2 combined to levels 3 and 4 combined to level 5. We conducted an exploratory Bayesian informed hypothesis test ANOVA in JASP [129,130], including exactly these predictions. The data provided most evidence for the *L*1 = *L*2 > *L*3 = *L*4 > *L*5 ordering (see tables 7 and 8). This suggests that there is some hint of a general negative relation between education and paranormal beliefs, but not sufficiently strong to reach convincing evidence in a linear regression analysis. Note that this analysis is highly exploratory and post hoc, so we recommend caution in interpreting these results.

**Table 7.** Hypothesis legend.

	Hypothesis
H1	education1 = education2 = education3 = education4 = education5
H2	education1 > education2 > education3 > education4 > education5
H3	education1 = education2 = education3 = education4 > education5
H4	education1 = education2 > education3 = education4 > education5

**Table 8.** Bain ANOVA.

	BF.u	BF.c	PMPa	PMPb	PMPc
H1	$6.043\times 10^{-6}$	$6.043\times 10^{-6}$	$2.245\times 10^{-9}$	$2.245\times 10^{-9}$	$2.245\times 10^{-9}$
H2	7.314	7.738	0.003	0.003	0.003
H3	1049.994	1049.994	0.390	0.390	0.390
H4	1633.818	1633.818	0.607	0.607	0.607
Hu				$3.715\times 10^{-4}$	
Hc	0.945				$3.511\times 10^{-4}$

*Note*. BF.u denotes the Bayes factor of the hypothesis at hand versus the unconstrained hypothesis Hu. BF.c denotes the Bayes factor of the hypothesis at hand versus its complement. PMPa contains the posterior model probabilities of the hypotheses specified. PMPb adds Hu, the unconstrained hypothesis. PMPc adds Hc, the complement of the union of the hypotheses specified. All PMPs are based on equal prior model probabilities.
